# Are We Ready to Reclassify Crohn’s Disease Using Molecular Classification?

**DOI:** 10.3390/jcm12185786

**Published:** 2023-09-05

**Authors:** Shahed Kamal, Nikita Parkash, William Beattie, Britt Christensen, Jonathan P. Segal

**Affiliations:** 1 Department of Gastroenterology, Northern Hospital, Epping, Melbourne VIC 3076, Australia; 2 Department of Gastroenterology, Royal Melbourne Hospital, Parkville, Melbourne VIC 3052, Australia; 3 Department of Gastroenterology, The University of Melbourne, Parkville, Melbourne VIC 3010, Australia

**Keywords:** Crohn’s disease, biomarkers, recurrence of disease, perianal disease, unmet needs

## Abstract

Crohn’s disease (CD) is a type of inflammatory bowel disease. The number of IBD cases worldwide was estimated to be 4.9 million in 2019. CD exhibits heterogeneity in clinical presentation, anatomical involvement, disease behaviour, clinical course and response to treatment. The classical description of CD involves transmural inflammation with skip lesions anywhere along the entire gastrointestinal tract. The complexity and heterogeneity of Crohn’s disease is not currently reflected in the conventional classification system. Though the knowledge of Crohn’s pathophysiology remains far from understood, the established complex interplay of the omics—genomics, transcriptomics, proteomics, epigenomics, metagenomics, metabolomics, lipidomics and immunophenomics—provides numerous targets for potential molecular markers of disease. Advancing technology has enabled identification of small molecules within these omics, which can be extrapolated to differentiate types of Crohn’s disease. The multi-omic future of Crohn’s disease is promising, with potential for advancements in understanding of its pathogenesis and implementation of personalised medicine.

## 1. Introduction

### 1.1. Crohn’s Disease

Crohn’s disease (CD) and ulcerative colitis (UC) are chronic inflammatory bowel diseases (IBD). The number of IBD cases worldwide was estimated to be 4.9 million in 2019 [1].

CD exhibits heterogeneity in clinical presentation, anatomical involvement, disease behaviour, clinical course and response to treatment [2]. The classical description of CD involves transmural inflammation with skip lesions anywhere along the entire gastrointestinal tract [3]. However, the most frequent site of CD pathology is in the ileocecal area, with up to a third of cases in females consisting of isolated colonic involvement [2,4]. Moreover, there is variation in the frequency of symptoms among patients [5,6]. The natural history of the disease also varies, with over half of patients with CD developing complications including strictures, fistulas, abscesses, perforation, obstruction, peritonitis and perianal disease [7,8,9].

These factors highlight the complexity of CD. Thus, there is a need for a classification system for CD that will allow prognostication, guide clinical decisions, inform patients of their disease and stratify patients for research and clinical studies. In this review, we first discuss the current classification of CD and its pitfalls. We then explore the advances in the molecular classification of CD, which include serological markers and the omics approaches.

### 1.2. Current Classification of CD

The current basis for prognosis and clinical management decisions in CD is the phenotypic classification at the time of diagnosis [3]. The Rome classification was developed in 1991 but was deemed inappropriate for clinical use and was replaced by the Vienna classification in 1995 [10]. The Montreal classification developed in 2005 modified the Vienna classification, and it is currently used by clinicians and researchers to classify CD (Table 1) [11].

The Montreal classification recognised the role of serological or genetic markers in CD classification but was not yet justified for recommendation at the time [12]. Currently, serum C-reactive protein (CRP) and faecal calprotectin are biomarkers that are used clinically to monitor disease activity [13,14].

### 1.3. Problems with Current CD Classification Systems

Early population-based studies suggested that the Montreal classification was a valid tool for predicting disease prognoses [15,16]. However, further studies have revealed several issues that hinder its clinical utility. These include the emphasis on the clinical phenotype over the underlying biology of the disease, poor predictive performance and poor reliability.

The first issue of the Montreal classification is its limitations in describing the dynamic and diverse nature of the disease. Several studies described the evolution of disease behaviour over time [2,17,18,19]. The current classification system also assumes a cumulative progression of the disease and does not take into account the remitting nature of the course of the disease in some patients over time (rolling phenotype) [20]. More importantly, a phenotype-based classification does not take into account the biological drivers of the disease. The current classification scheme does not explain CD complexity and the wide variation in clinical presentation, disease progression and treatment response [21]. Indeed, molecular subtypes that are clinically relevant have been identified for CD [22,23].

Second, studies have demonstrated the poor predictive performance of the Montreal classification, while other measures perform better. In a retrospective study, classifying patients into colon-involving or non-colon-involving was a better predictor for surgery or medical therapy in patients with CD than the Montreal classification [24]. Moreover, the Montreal classification did not distinguish subpopulations in terms of serum inflammatory markers, need for surgery, steroid use or infliximab use. In another report, the Montreal classification correlated with the Crohn’s Disease Endoscopic Index of Severity (CDEIS) for the disease behaviour parameter, while none of the parameters correlated with the D’Haens histopathological classification [25]. Moreover, there are differences in the prognosis of subphenotypes in the Montreal classification. For example, under the L4 classification, the prognosis is better in patients with L4-esophagogastroduodenal (EGD) involvement than without EGD involvement [26]. Other demographic factors such as sex or smoking can by themselves be predictors of disease outcome [27].

Lastly, one of the pitfalls of the Montreal classification is its reliability. A previous report determined the performance of the Montreal classification when used by practising gastroenterologists, gastroenterologists in training and IBD nurses [28]. Overall, the classification system showed acceptable performance in terms of correct answers provided by the participants. However, the IBD nurses made more errors in scoring the age of onset and upper gastrointestinal disease. Indeed, inter-observer agreement was lower for these parameters. Furthermore, correct scoring using Montreal classification did not correlate with improved assessment of disease severity or years of experience. In a population-based study, using the national registry to validate the Montreal Classification led to inconsistent results, except for CD patients with perianal involvement [29].

The weaknesses of the Montreal classification pose a challenge to our complete understanding of CD. Thus, the use of novel biomarkers for the molecular classification of CD is a promising alternative that can offer superior predictive performance, allow a personalised approach to management and elucidate underlying biological mechanisms that can guide the development of targeted treatment. The aim is to apply molecular classification to CD, as has been successfully achieved in other complex diseases such as cancer [30].

## 2. Serological Markers

The presence of antibodies against autoantigens and microbial antigens has long been known in CD [31]. Although these serological markers offer moderate accuracy for diagnosing CD, these antibodies can be useful for stratifying patients according to clinical outcomes.

Antibodies against fungal and bacterial pathogens have been documented early on in the serum of patients with CD. High levels of anti-*Saccharomyces cerevisiae* antibodies (ASCA) have been found to be associated with early age of onset, development of strictures or fistula, severe disease and the need for surgery [32,33,34,35]. Compared with other serological markers reported in the literature, ASCA is superior for diagnosing CD, with a sensitivity of 51–63% and specificity of 77–96% [36]. Anti-*Pseudomonas fluorescens* antibodies (anti-I2) may be linked with stenosis and surgery [35,37].

Antibodies against microbial protein or carbohydrate antigens have also been recognised in CD. The anti-outer membrane protein C of the *Escherichia coli* antibody (anti-OmpC) is associated with perforating disease and the need for surgery, while anti-flagellin antibodies (anti-CBir1) are associated with penetrating and stenosing disease [32,35,37,38]. Anti-mannobioside carbohydrate antibodies (AMCA), anti-chitobioside carbohydrate antibodies (ACCA) and anti-laminarobioside carbohydrate antibodies (ALCA) are associated with stricturing and penetrating disease [32,33]. Anti-OmpC, AMCA, ACCA and ALCA have sensitivities of 27%, 12–27%, 11–17% and 15–26%, respectively, and specificities of 75%, 92–100%, 80–98% and 89–98%, respectively, for diagnosing CD [36].

The perinuclear antineutrophil cytoplasmic antibody (pANCA) autoantibody is found in patients with IBD but appears to be more frequent in patients with UC than CD [35,39]. Nevertheless, higher pANCA levels in CD patients suggest colonic location, and they were positively associated with later age of onset and UC-like behaviour but negatively associated with stenosis and the need for surgery [34,36,37].

Important caveats in interpreting these reports include research design, inconsistent methodological details and conflicting results. A meta-analysis found that the stability of these antibodies over time was evaluated by only a few studies and at different specific time points [36]. Moreover, many antibodies shown to be associated with CD complications and progression have not been validated in more recent studies [36]. Most of these studies were cross-sectional in design and measured only at one time point [36]. Furthermore, whether these serum titres were measured before treatment or in response to therapy was not explicitly mentioned [36].

Studies on serological markers in CD suffer from methodological issues, limiting their generalisability for clinical use. Furthermore, their poor diagnostic sensitivity could pose a challenge in their application. Thus, prospective studies that will evaluate the association of serological markers with clinically relevant outcomes are warranted before these can be used in the molecular classification of CD.

## 3. Omics Approaches for the Molecular Classification of CD

**Figure 1:**
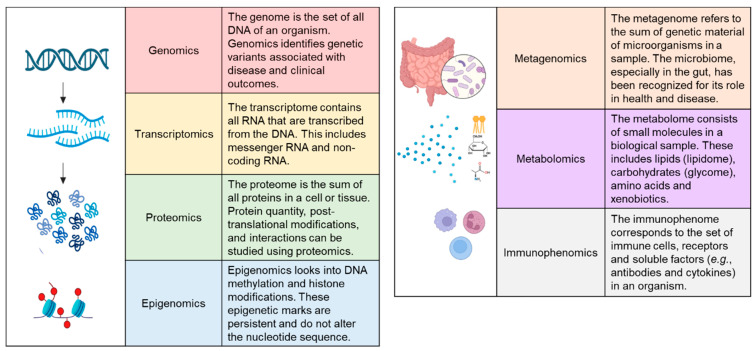
The omics approach. Created with BioRender.com.

The rapid development of omics technologies has revolutionised the characterisation of diseases. Genomics, transcriptomics, proteomics, epigenomics, metagenomics, metabolomics, lipidomics and immunophenomics have allowed deeper phenotyping for diagnostic classification, developing personalised medicine and predicting clinical outcomes (Figure 1). In the next sections, we review each of these omics technologies and their utility in classifying CD.

## 4. Genomics

Whilst the genetic underpinnings of Crohn’s disease remain incompletely understood, a number of genetic predispositions have been identified. Early studies identified that up to 80% of siblings with Crohn’s disease had concordance for their phenotype of disease [40]. One of the early genes identified was NOD2/CARD15 on chromosome 16 [41,42], which was analysed using polymerase chain reaction (PCR) sequence-specific primers to determine certain mutant alleles that were predictive of ileal Crohn’s disease but not colonic disease [43]. Despite knowledge of the associated between NOD2/CARD15 mutations and Crohn’s (especially ileal Crohn’s), the true extent of the importance of the NOD2/CARD15 gene in Crohn’s disease remains incompletely understood and has yet to permeate into clinical practice.

Over time, numerous other potential gene loci have been identified that predict Crohn’s disease occurrence and behaviour. A meta-analysis of genome-wide association studies (GWAS) identified 163 genes associated with Crohn’s disease, being implicated in nearly all aspects of cellular function including RNA processing, lipid metabolism, oxidative stress, xenobiotic metabolism and G-protein coupled receptor (GPCR) signalling [44]. A recent GWAS corroborated more than 200 gene loci contributing to the risk of CD identified previously while identifying 25 novel loci [45]. Among these novel loci, variants in *SLAM8*, a receptor that negatively regulates inflammation, and *RORC*, a transcriptional regulator of Th17 cell differentiation, were identified as most likely causal for CD. Other genes that may be likely to contribute to CD pathogenesis are the genes encoding for a phospholipase (*PLCG2*) and integrins (*ITGA4*, *ITGAV*, *ITGB8* and *ICAM1*). A recent large-scale GWAS analyzing sequencing data from 30,000 patients with CD and 80,000 controls was able to confirm the genetic significance of *NOD2* and identified new CD-associated genes involving autophagy and mesenchymal cells [46]. More recent evidence suggests that these patterns may differ between ileal and colonic Crohn’s, leading us closer to delineating the genotype for different clinical subtypes of Crohn’s disease [23].

Chip-based genomics, such as that offered by the Immunochip platform, has allowed large throughput analysis of the association between autoimmune disease and genetic polymorphisms by analysing single-nucleotide polymorphisms (SNPs) identified in GWAS [47]. Using this technique, the genetic profile of ileal Crohn’s has been determined to be distinct from colonic Crohn’s as it is from ulcerative colitis. This technique has reinforced the association between NOD2 and ileal Crohn’s as well as that of MHC and colonic Crohn’s (identifying the HLA alleles DRB1*01:03 and BRB1*07:01). A third gene, MST1 3p21, carrying SNPs with a signal for disease, was identified using this technique and was associated with an earlier age of onset [40].

Given the multifactorial nature of Crohn’s, no single genetic variant can predict the risk of disease or its clinical course. Polygenic risk scores provide a means to aggregate the genes implicated in Crohn’s disease to then potentially predict the likelihood of an individual developing the disease [48]. Acknowledging the diverse ethnic populations affected by Crohn’s disease and the variability in their respective implicated genetic loci, a recent study combined polygenic risk scores from European, African-American and Ashkenazi Jewish reference case-control studies to successfully improve the prediction of disease in these cohorts [49]. Another study, which evaluated the polygenic risk scores of two independent IBD cohorts, found an association between the composite genetic risk of Crohn’s and fibrostenotic disease, even after the exclusion of NOD2, MHC and MST1 [50]. A similar association was found with the risk of ileocaecal resection, including after removal of the known susceptibility loci, implying that patients with a high polygenic risk score were more likely to develop fibrostenotic disease and undergo ileocaecal resection. This study suggests a potential role of genomics in disease classification and course.

Ultimately, the long-term goal for achieving an accurate molecular classification of Crohn’s will be to identify genotypes that predispose to particular Crohn’s phenotypes (including extent location and the presence of a penetrating disease). A recent GWAS comparing CD patients with good and poor prognoses identified four gene loci that were not previously associated with disease susceptibility [51]. These genes were *FOXO3*, *XACT*, a locus upstream to IGFBP1 and the MHC locus. This study suggests that the genes associated with disease risk are distinct from those that determine disease prognosis. Larger studies in diverse populations are required to verify these prognosticator genes and translate these genetic data to improve clinical outcomes.

## 5. Transcriptomics

Transcriptomics aims to achieve a molecular classification of Crohn’s disease by analysing RNA expression in tissue or immune cells (known as the transcriptome). Early studies identified differences in RNA expressed in colonic tissue between controls, patients with Crohn’s disease and those with ulcerative colitis using a microarray [52]. Similarly, transcriptomic differences were identified in circulating blood mononuclear cells between patients with Crohn’s disease and ulcerative colitis [53]. These studies identified patterns across several diseases that distinguish patients with and without Crohn’s disease, but they could not identify individual genetic markers for use in clinical practice.

More recently, differences in the expression of cytokines have been identified between inflamed and non-inflamed bowel tissue in the same patient with Crohn’s disease, including chemokine ligand 1 (CXCL1, a chemokine with neutrophil attracting activity), chemokine ligand 20 (CCL20), C4b binding protein (C4BBP, a protein that controls the complement cascade) and interleukin 1 receptor antagonist (IL1RN) [54,55,56]. Ultimately, the aim is to analyse tissue from individuals with Crohn’s disease and predict the disease course and response to treatment. One recent study utilised single-cell RNA sequencing (scRNA-seq) technology to predict the response to anti-TNF therapy by uncovering a unique cellular footprint comprising IgG plasma cells, inflammatory mononuclear phagocytes, activated T cells and stromal cells [57].

Transcriptomics analysis has also permitted a better understanding of the role that fibrosis plays in the pathogenesis of Crohn’s. The assessment of ileal and colonic tissues has revealed specific genes regulating myofibroblast activation, namely CHMP1A, TBX3 and RNF168. The same study noted differences in gene expression between the ileal and colonic tissue [58], supporting existing genomic studies that highlight the differences between small- and large-bowel Crohn’s [23,43]. Transcriptomic analysis of fibroblast activity can also assess the response to specific medications for Crohn’s, demonstrating that infliximab does not induce a pro-fibroblast transcriptional response despite increased transforming growth factor β_1_ (TGF-β_1_) expression [59].

The development of organoids in bowel tissue from patients with IBD has permitted in vitro transcriptomic analysis in a more physiologically relevant cellular environment [60,61]. In particular, organoids derived from the ileal tissue of patients with Crohn’s disease have been demonstrated to have 90% transcriptomic congruence with the ileal tissue it was derived from, with the benefit of displaying a number of features and functions of intestinal epithelium, such as barrier function, differentiation and self-renewal [62]. Similarly, the colonic organoids derived from both patients with Crohn’s and ulcerative colitis were found to demonstrate a similar inflammatory phenotype to the parent tissues [61]. Ultimately, the use of organoids aims to improve our understanding of the cellular and molecular response to therapeutics, with the eventual goal of providing personalised medicine [63].

## 6. Proteomics

Proteins serve a variety of functions in different biological processes, including structural, enzymatic, transport, immunity and cell signalling [64]. The proteome may be considered to be more dynamic than the genome while being stabler than the transcriptome. Thus, the proteome more accurately reflects cellular function and may offer a promising approach in the molecular classification of CD.

Several studies have demonstrated that the proteome can be used to diagnose IBD, differentiate CD from UC or intestinal tuberculosis and even estimate the risk of developing CD [65,66,67,68,69,70,71]. A more daunting yet relevant task is to utilise proteomic profiles as the basis for classifying CD. This approach can enable a more precise stratification of patients with CD to better guide treatment, monitor disease activity and predict clinical outcomes.

One of the earliest studies that explored the proteome in CD aimed to predict treatment response to infliximab [72]. Surface-enhanced laser desorption/ionisation time of flight mass spectrometry (SELDI-TOF MS) was used to determine the serum proteome pre- and post-treatment. Predictive modelling based on their MS data resulted in a sensitivity of 78.6%, specificity of 80.0% and accuracy of 79.3% for predicting treatment outcomes [72]. Further analysis allowed the identification of platelet aggregation factor 4, which was increased in the non-responding patients [72]. This study implicates the role of platelet metabolism in therapeutic responses to an anti-TNF-α antibody.

In another early study, the proteomic profiles in peripheral blood mononuclear cells were analysed using 2D gel electrophoresis (2DGE) and tandem MS [73]. Eleven proteins were successfully identified to be different between CD and UC patients. Among these, two proteins were able to predict disease activity and CRP levels.

Serum proteome can also be used to distinguish stricturing from non-stricturing CD. Analysis of the serum of adult and paediatric patients with CD using LC-MS revealed 16 proteins that are useful for differentiating CD with stricture from CD without stricture [74]. This set of proteins includes alpha-2-macroglobulin, L-lactate dehydrogenase B chain, cathepsin D, apolipoprotein B-100, serum albumin and ceruloplasmin. Partial least squares discriminant analysis showed that this approach is 70% accurate when using the peptide database and up to 80% accurate when using the protein database [74].

Stool biomarkers can also be analysed to determine the proteome. Thus, 2DGE and matrix-assisted laser desorption/ionisation time-of-flight/time-of-flight mass spectrometry (MALDI-TOF/TOF MS) revealed 21 proteins correlated with intestinal inflammation in CD [75]. Chymotrypsin C, gelsolin and Rho GDP-dissociation inhibitor 2 (RhoGDI2) were significantly correlated with disease severity and were more sensitive and specific than faecal calprotectin in diagnosing CD [75].

Immunoassays can be employed to quantify proteins in a targeted manner. A previous study proposed the endoscopic healing index (EHI), which is a score based on the levels of 13 serum proteins [76]. These proteins were involved in angiogenesis (ANG1 and ANG2), inflammation (CRP and SAA1), immunomodulation (IL7), matrix remodelling (EMMPRIN, MMP1, MMP2, MMP3 and MMP9), cell growth (TGFA) and cell adhesion (CEACAM1 and VCAM1). The performance of EHI was better than CRP and on par with faecal calprotectin [76]. However, EHI was not able to distinguish remission and active disease across disease locations and behaviours.

An emerging method for studying the proteome is the proximity extension assay (PEA). In this method, antibody pairs that bind to the same antigen allow oligonucleotide hybridisation and protein identification. This approach led to the identification of 15 proteins in the TNF-independent pathways that predict treatment escalation [77]. In the same study, the parameters of the Montreal classification, including non-B1 disease behaviour and perianal disease, were not associated with treatment escalation. This highlights the superior performance of proteomics in classifying CD and predicting disease outcomes [77].

The PEA has also been used along with other omics approaches to determine association with disease remission. Six proteins were associated with endoscopic remission, while CASP8 showed a different relationship with remission, depending on if the patient is in anti-cytokine or anti-integrin therapy [78]. A composite model integrating clinical, metagenomic, metabolomic and proteomic markers resulted in the optimal performance for predicting treatment response. In another study, disease activity was associated with the levels of four proteins identified through PEA [79]. Protein quantitative trait loci (pQTL) analysis was employed to determine the effect of genetics on the plasma protein level. In the patients with CD, 23 pQTLs were identified.

These studies provide a proof of concept of the utility of proteomic technologies in classifying CD according to treatment response and clinical outcomes. A caveat for interpreting proteome studies is the wide range of techniques, experimental protocols and analytical tools used to generate the proteomics profiles in patients [80,81]. This makes corroboration of proteomic findings difficult, warranting further studies to ensure the validity and replicability of these methods. An alternative approach would be to design targeted proteome panels to quantify pre-identified proteins that are strongly associated with CD prognosis.

## 7. Epigenomics

The epigenome refers to the totality of the stable but dynamic mechanisms of gene regulation without changes in the nucleotide sequence [82,83]. DNA methylation, histone modifications and non-coding RNA are well-studied epigenetic changes that regulate gene expression by modifying access to the DNA or mRNA. The epigenome is thought to facilitate the interaction between genes and the environment, resulting in diverse phenotypes in cells or organisms with identical genomes. The influence of environmental factors, such as smoking, diet, physical activity and vitamin D supplementation, has been studied in clinical and preclinical studies on CD [84,85,86].

In an early epigenome-wide association study (EWAS), 50 differentially methylated regions (DMRs) were identified in whole blood samples of female adult and paediatric patients with CD [87]. These DMRs were related to immune-related genes such as *MAPK13*, *RIPK3* and *IL21R* and were also enriched near the gene loci previously identified in GWAS studies, such as *TNF* and *NOD2* [87]. The DNA methylation profile in the blood sample was demonstrated to diagnose CD with a sensitivity of 71% and specificity of 83% [87]. Later studies have identified a set of DNA methylation patterns observed across studies that analysed either the bowel or peripheral blood samples of patients with CD [88,89]. These include hypomethylation of *VMP1*, *TNF* and *SPI1* and hypermethylation of *TNFSF4* and *RPS6KA2* [90,91,92].

Several efforts have been made to compare the profile of subpopulations of patients with CD. EWASs on colonic and ileal samples from patients with CD were able to identify DMRs associated with fibrostenotic disease [93,94]. These DMRs were validated with transcriptomic data and were associated with genes related to fibrosis. In patients who underwent colonic resection, five DMRs were associated with disease recurrence, including a locus associated with *RPS6KA2* [95]. In another report, chromatin access and gene expression profiles were clustered into two classes that may also be used to classify CD patients [23].

Recent studies have explored the prognostic role of the epigenome with regard to treatment outcomes. In paediatric patients, DNA methylation patterns in mucosal biopsies can be used to predict the need for biologics and treatment escalation, with a sensitivity of up to 75% and specificity of 100% [96]. However, the transcriptomic data outperformed the methylomic data for prognosticating outcomes. In a study on children and adults with IBD, the methylome signature of a panel composed of *TAP1*, *TESPA1* and *RPTOR* predicted a fivefold increase in the risk of treatment escalation [91].

Epigenetic panels are currently being evaluated for predicting treatment response to specific biologics for CD. CpG panels consisting of 100, 25 and 68 loci in peripheral blood can predict the clinical and endoscopic response to adalimumab, vedolizumab and ustekinumab, respectively, with accuracies of 73%, 88% and 94%, respectively [97,98]. For vedolizumab, a 23-CpG panel can predict deep remission with an accuracy of 75% [98].

The suitability of using epigenetic markers for CD diagnosis and prognosis in adult patients is well supported by the stability of a subset of DMRs over time [96,99]. Across a median period of 7 years, 5% of the DMRs in the peripheral blood were stable, including 22 CD-associated genes and HLA genes [99]. However, in the paediatric patients with CD, changes in the blood methylome were reversed within 1–3 years of treatment [100]. This occurred along with a decrease in inflammatory marker levels but without regard to disease progression [100]. This implies that in paediatric patients, epigenomic signatures may be a consequence of inflammation due to CD rather than contributing to disease pathogenesis, limiting its use for diagnosis and prognosis.

An advantage of using epigenomic markers for the classification of CD is the possibility of using peripheral blood, which is more accessible compared with biopsy samples. While DNA methylation profiling has great potential for CD diagnostics and prognostics, future studies with appropriate design and power are needed to evaluate the robustness of previous findings, especially to evaluate the epigenome independent of transcriptomic data.

## 8. Metagenomics

Changes to the gut microbiome are a well-known key driver of the pathogenesis of Crohn’s disease. In healthy subjects, the gut is populated by trillions of bacteria, protozoa, fungi and viruses, being predominated by five bacterial phyla: *Bacteroidetes, Firmicutes, Actinobacteria, Proteobacteria* and *Fusobacteria* [101]. Perturbations in the composition of the gut microbiota result in a decrease in diversity and an imbalance in protective and pathogenic organisms, promoting an inflammatory response [102]. The study of the microbiome has expanded exponentially through the integration of 16S rRNA gene sequencing, providing a rapid, culture-free method for sequencing the microbiome [103].

Crohn’s disease has been distinguished from ulcerative colitis at the microbiome level using high-throughput DNA sequencing of faecal samples [104]. The increased degree of perturbations in the gut microbiome is evident, with a lower relative abundance of the microorganism groups *Faecalibacterium*, *Peptostreptococcaceae*, *Anaerostipes*, *Methanobrevibacter*, *Christensenellaceae* and *Collinsella* in Crohn’s disease compared with ulcerative colitis and a higher relative abundance of *Fusobacterium* and *Escherichia*. *Fusobacterium* is the genus most consistently associated with Crohn’s disease and may serve as a potential biomarker when a diagnosis of Crohn’s disease is debated [105].

A recent study of the gut microbiome of patients with terminal ileal, small-bowel and colonic subtypes of Crohn’s disease revealed that terminal ileal disease, whilst being enriched for *Faecalibacterium,* was largely indistinguishable from the microbiota diversity of healthy controls [106]. Conversely, the colonic and small-bowel subtypes were enriched for the opportunistic pathogens *Streptococcus* and *Burkholderia* as well as *Escherichia* and *Acinetobacter*, respectively. Significant differences in the microbiome of ileal versus colonic Crohn’s disease have been mirrored in other studies [107,108].

Changes in the microbiome can also be used for risk stratification for complications of Crohn’s disease. A study of the microbiota of children with Crohn’s disease was conducted by analysing ileal and rectal stool samples [109]. *Ruminococcus* was associated with stricturing complications, and *Veillonella* was associated with penetrating complications of Crohn’s disease [109]. These results could be further validated in an adult population and used for a risk stratification tool for complications from Crohn’s disease.

Although our understanding of the microbiome in Crohn’s disease is quickly evolving, the results are not yet proven to be clinically significant, with attempts at manipulating the microbiome such as faecal transplants, diets and probiotics falling short of significance [110,111].

## 9. Metabolomics

The metabolome encompasses small molecules (molecular weight less than 1500 Daltons) in biological samples [112,113]. Metabolomic analyses can be performed on samples that are easy to access, including blood, stool, urine and saliva [114]. Depending on the sample, the metabolome reflects cellular metabolism, microbiome metabolism, diet and xenobiotics [115]. Nuclear magnetic resonance (NMR) spectroscopy and mass spectrometry (MS) are the most powerful analytical techniques for determining metabolomic profiles, particularly in the context of disease [113].

An early study carried out to characterise the metabolome in IBD used NMR to analyse faecal extracts from patients with CD or UC and healthy controls [116]. The patients with IBD showed a lower abundance of the short-chain fatty acids (SCFA) butyrate and acetate and the amines methylamine and trimethylamine when compared with the controls. This depletion was more prominent in the patients with CD than those with UC. Furthermore, glycerol was more highly enriched in the stool samples of the patients with CD than those with UC [116]. These differences in the metabolomic profiles between CD and UC were thought to indicate the increased severity and anatomical extent of pathological inflammation in CD.

Studies on the metabolome in IBD have increased and been reviewed recently [115,117]. Patients with IBD showed increased metabolite markers of inflammation (arachidonic acid derivatives), changes in cellular metabolism (tricarboxylic acid cycle intermediates) and perturbations in the gut microbiome (reduced SCFA, reduced hippurate and changes in bile acid levels) [117]. Furthermore, differences in metabolomic profiles have been observed between CD and UC and among the different phenotypes of CD [115]. However, most of these studies had small sample sizes and were limited in the patient data collected.

A recent study used an untargeted metabolomics approach in a larger patient sample and evaluated the correlation of faecal metabolite levels with gut bacteria, diet and genetics [118]. Compared with the controls, the CD patients differed in terms of the abundance of 324 metabolites. The CD patients showed increased sphingolipids and ethanolamines, which could be related to inflammation. About 60% of these potential biomarkers were shared between CD and UC, with a decrease in vitamins and SCFA seen in both diseases [118]. The ratio of lactosyl-N-palmitoyl-sphingosine (d18:1/16:0) and L-urobilin levels was proposed as a biomarker for diagnosing IBD [118]. Interestingly, dietary intake and genetics did not greatly impact the faecal metabolome.

Similar findings were seen in children with CD. In a study performed with paediatric CD patients, the metabolites N-acetyl glycoprotein, glycerol and phenylalanine were correlated with plasma CRP [119]. The metabolite levels were also correlated with inflammatory genes, including *IL12B*, *IL12RB2*, *IL6* and *NFKB*.

The faecal metabolome has been closely linked to the gut microbiota, with correlations observed between specific metabolites and the presence of specific bacterial taxa [118]. Bacterial abundance accounted for more than 40% of the variation in metabolite levels, compared with 20% accounted for by dietary factors [118].

These findings were consistent with a study conducted with an Asian population [120]. In this study, metabolites that were sensitive and specific to IBD included 6,7,4′-trihydroxyisoflavone, thyroxine 4’-o-beta-d-glucuronide, trichostachine and normorphine. Further analysis revealed 52 flora-metabolite pairs that were significantly correlated and could be used as biomarkers for diagnostics or drug development [120].

While metabolomics approaches can potentially be used for CD diagnosis and prognosis, more studies are warranted to validate previous findings and determine the ideal sample, analytical method and metabolites of interest.

## 10. Lipidomics

Lipidomics, a subset of metabolomics, is the study of lipids and is an emerging branch of omics in the study of IBD pathogenesis and diagnosis.

Lipids have a variety of functions in the body, including forming cell membranes, insulating neurons and serving as chemical messengers and as an energy source [121]. The large number of analysable lipids can be broadly grouped into eight categories: fatty acids, glycerolipids, glycerophospholipids, sphingolipids, saccharolipids, polyketides, sterols and prenols [122]. Both endogenous and exogenous lipids are suggested to be implicated in the pathogenesis of intestinal inflammation associated with IBD [123].

Lipids can be analysed directly from biological samples (e.g., serum, plasma, tissue or faeces) or following extraction with various solvents. Liquid-liquid and solid-phase extractions are the most common preparation techniques in lipidomics, with the former largely used for untargeted studies and the latter used for targeted studies [124]. Most preliminary lipidomics studies are untargeted to allow for a comprehensive unbiased analysis. Lipidomic analysis methods are similar to those employed for proteomics; samples are analysed primarily with advanced mass spectrometry technologies [125].

Several studies have employed the use of lipidomics to differentiate between healthy controls, CD patients and UC patients by identifying lipidomic markers and their relative concentrations in these population groups [126,127,128,129]. Other studies have further analysed lipid profiles generated through lipidomics, using pathway analysis methods to investigate altered metabolic pathways associated with CD [130,131,132,133,134]. Numerous lipids were implicated in the various studies, largely glycerophospholipids, sphingolipids and fatty acids. There are currently no studies further classifying CD into subtypes using lipidomics. The altered lipid profiles and pathways have the potential to guide future diagnostics in CD and IBD, but detailed targeted lipidomic studies are required to address this deficit.

Lipidomics has also been used for identifying lipid profiles and dysregulated pathways associated with fatigue, a symptom present in nearly 80% of patients with active IBD and 50% of patients with inactive IBD [134]. Plasma from quiescent UC and CD patients (further classified as fatigued and non-fatigued) were analysed using ultraperformance liquid chromatography time-of-flight mass spectrometry (UPLC-TOFMS). The analysis revealed significantly decreased levels for eight lipids in patients with IBD and fatigue, and further pathway analysis suggested dysregulation of the arachidonic acid and glycerophospholipid metabolisms and the sphingolipid pathway [134]. Fatigue, the assessment of which is largely subjective, had previously been associated with multiple risk factors, though no clear cause had been identified.

The application of lipidomics in IBD treatment and remission is in its preliminary stages, with studies primarily limited to experimental mice studies [135,136]. A lipidomic analysis of mouse serum was performed in mice that received dextran sodium sulfate in their drinking water to induce intestinal inflammation as a replica of IBD [135]. The mice then received a period of normal drinking water to promote the intestinal healing process. The use of LC-MS demonstrated reduced levels of arachidonic acid (a precursor of prostaglandin F_2α_), 19H-PGF_1α_ (a metabolite of prostacyclin) and 20H-PGF_2α_ (a metabolite of prostaglandin F_2α_), which suggested resolving inflammation. Increased levels of an active metabolite of resolvin D1 (a lipid mediator associated with anti-inflammatory properties) and decreased levels of its precursor (DHA) and the precursor of resolvin E (EPA) suggested mucosal healing [135]. The mice were supplemented with exogenous fish oil, and DHA and EPA accelerated mucosal healing, suggesting a potential role for exogenous pro-healing lipids in maintaining remission in IBD.

As a newer avenue of biomarker detection in IBD compared with other omics, such as proteomics, only limited studies exist on lipidomics in CD. These studies have identified a large variety of lipids associated with CD. However, only a few studies have overlapped in the analysis of specific lipids [137]. There is also no consensus on the best specimen for analysis in the current literature—blood, tissue or faeces—which have varying levels of invasiveness and ease of collection [138]. Further targeted lipidomic studies and clinical trials are required as a next step to identify the clinical significance of these lipids and their use in personalised medicine.

## 11. Immunophenomics

Although the exact pathophysiology of CD is unknown, a dysregulated mucosal immune response to the gut microbiome is a driving factor in IBD [139]. Therefore, studying the immunophenome may provide vital information for characterising CD and its subtypes and predicting which patient populations would respond best to immune-related treatments.

Immunophenotyping in IBD can be performed either at the local (tissue) level or at the peripheral (blood) level. Studies comparing the levels of immune cells, their receptors and associated cytokines from the intestinal mucosa of healthy controls, UC and CD patients have identified patterns of expression between the two pathologies [140,141]. These include elevated levels of IFN-γ and decreased levels of CD3 and T cell receptors alpha/beta and gamma/delta in CD patients compared with UC patients and the healthy controls.

A recent study explored alterations in the peripheral immunophenome in UC and CD patients compared with healthy controls [142]. Analysis of blood via fluorescence-activated cell sorting showed that whilst there were few cell types implicated in both UC and CD, the immunophenome was largely distinct; CD had higher proportions of neutrophils, Th1, Th17, memory CD4 T cells and CD 27^-^ B cells and lower proportions of overall T cells and CD4+CD8+ T cells [142].

In addition to differentiating between UC and CD, immunophenotyping has a role in the location subclassification of CD: ileal versus colonic disease. Two studies utilising flow cytometry to compare the intestinal mucosa immune cells in CD patients found an increase in Th17 cells in the ileum but not the colon and an increase in Th1 cells in both the ileum and colon [140,143].

Immunophenotyping in CD complications may uncover potential biomarkers for disease course and therefore drug targets in CD [144]. In the aforementioned study, immunophenotyped blood samples of CD patients found that elevated effector memory CD4 and CD8+ T cells were associated with stricturing and penetrating CD and decreased naïve CD4+ T cells were associated with increased CD duration and number of surgeries [142]. Lymphocytic populations in the intestinal mucosa of IBD patients were analysed in another study using flow cytometry, which found that CD patients who later developed stricturing or penetrating disease had higher levels of CD4+ and regulatory T cells compared with those with uncomplicated CD [145].

Methods previously used to identify a transcriptional signature of CD8+ T cells that could prognosticate ANCA-associated vasculitis and systemic lupus erythematosus were recently applied to IBD [146,147]. Whole genome transcriptional analysis of CD8+ T cells extracted from the peripheral blood of Crohn’s disease and ulcerative colitis patients at diagnosis revealed significant differences in gene expression in IBD patients, who subsequently experienced a more aggressive disease course than those who had a lower frequency of relapse and complications. Statistical modelling was used to identify a correlating transcriptional signature of the whole blood and then optimised into a multi-gene qPCR assay for ease of testing [148]. This transcriptional signature is currently being employed to prognosticate patients with newly diagnosed CD in the biomarker-stratified trial “PROFILE” [149]. The trial is promising within the realm of personalised medicine, aiming to investigate whether patients deemed to have a high risk of relapsing disease would benefit from conventional treatment versus a “top-down” approach to treatment—initial aggressive treatment with infliximab and a concurrent immunomodulator—by comparing outcomes between the risk-stratified groups.

## 12. Conclusions

The complexity and heterogeneity of Crohn’s disease is not currently reflected in the conventional classification system. Though the knowledge of Crohn’s pathophysiology remains far from understood, the established complex interplay of the omics—genomics, transcriptomics, proteomics, epigenomics, metagenomics, metabolomics, lipidomics and immunophenomics—provides numerous targets for potential molecular markers of disease. Advancing technology has enabled the identification of small molecules within these omics, which can be extrapolated to differentiate types of Crohn’s disease. These molecules have mainly been studied in isolation, leaving the scope for integrating technological methods and multi-omics analyses to account for the intersection of the omics in Crohn’s disease. Despite the promising advancements in technologies and omics databases, implementation of multi-omics analyses on the larger populations required for prospective studies and clinical practice comes with significant costs, challenges secondary to intersubject heterogeneity and logistical barriers due to sampling and standardisation [107]. The integration of multi-omics analyses has led to advances in the diagnosis and treatment of other diseases, such as breast cancer [150]. Identified strategies to address the barriers to wide-scale implementation of multi-omics include increasing the accessibility of databases of diverse patient cohorts with multi-omic measurements from various tissue types, improving the efficiency of testing by applying machine learning methods and increasing funding for multi-omics [151]. Once these limitations are overcome and advancements in analyses lead to growth and accessibility of the multi-omic Crohn’s disease database, we can move away from associations and to causations. The multi-omic future of Crohn’s disease is promising, with potential for advancements in understanding of its pathogenesis and the implementation of personalised medicine.

## Figures and Tables

**Table 1:** Vienna classification and Montreal classification of Crohn’s disease [12].

	Vienna Classification	Montreal Classification
Age at diagnosis	A1 below 40 y A2 above 40 y	A1 below 16 y A2 between 17 and 40 y A3 above 40 y
Location	L1 ileal L2 colonic L3 ileocolonic L4 upper	L1 ileal L2 colonic L3 ileocolonic L4 isolated upper disease *
Behaviour	B1 non-stricturing, non-penetrating B2 stricturing B3 penetrating	B1 non-stricturing, non-penetrating B2 stricturing B3 penetrating p perianal disease modifier ^†^

* L4 can be used as a modifier for L1–L3 when upper gastrointestinal disease is also present. † “p” is added to B1–B3 in the presence of concomitant perianal disease. y = years old.

## Data Availability

Not applicable.

## References

[1] Wang R., Li Z., Liu S., Zhang D. (2023). Global, Regional and National Burden of Inflammatory Bowel Disease in 204 Countries and Territories from 1990 to 2019: A Systematic Analysis Based on the Global Burden of Disease Study 2019. BMJ Open.

[2] Cho C.W., You M.-W., Oh C.H., Lee C.K., Moon S.K. (2022). Long-Term Disease Course of Crohn’s Disease: Changes in Disease Location, Phenotype, Activities, and Predictive Factors. Gut Liver.

[3] Lichtenstein G.R., Loftus E.V., Isaacs K.L., Regueiro M.D., Gerson L.B., Sands B.E. (2018). ACG Clinical Guideline: Management of Crohn’s Disease in Adults. Am. J. Gastroenterol..

[4] Subramanian S., Ekbom A., Rhodes J.M. (2017). Recent Advances in Clinical Practice: A Systematic Review of Isolated Colonic Crohn’s Disease: The Third IBD?. Gut.

[5] Perler B.K., Ungaro R., Baird G., Mallette M., Bright R., Shah S., Shapiro J., Sands B.E. (2019). Presenting Symptoms in Inflammatory Bowel Disease: Descriptive Analysis of a Community-Based Inception Cohort. BMC Gastroenterol..

[6] Singh S., Blanchard A., Walker J.R., Graff L.A., Miller N., Bernstein C.N. (2011). Common Symptoms and Stressors Among Individuals With Inflammatory Bowel Diseases. Clin. Gastroenterol. Hepatol..

[7] Hsu Y.-C., Wu T.-C., Lo Y.-C., Wang L.-S. (2017). Gastrointestinal Complications and Extraintestinal Manifestations of Inflammatory Bowel Disease in Taiwan: A Population-Based Study. J. Chin. Med. Assoc..

[8] Li Y., Chen B., Gao X., Hu N., Huang M., Ran Z., Liu Z., Zhong J., Zou D., Wu X. (2019). Current Diagnosis and Management of Crohn’s Disease in China: Results from a Multicenter Prospective Disease Registry. BMC Gastroenterol..

[9] Shivashankar R., Tremaine W.J., Harmsen W.S., Loftus E.V. (2017). Incidence and Prevalence of Crohn’s Disease and Ulcerative Colitis in Olmsted County, Minnesota From 1970 Through 2010. Clin. Gastroenterol. Hepatol..

[10] Gasche C., Scholmerich J., Brynskov J., D’Haens G., Hanauer S.B., Irvine E.J., Jewell D.P., Rachmilewitz D., Sachar D.B., Sandborn W.J. (2000). A Simple Classification of Crohn’s Disease: Report of the Working Party for the World Congresses of Gastroenterology, Vienna 1998. Inflamm. Bowel. Dis..

[11] Silverberg M.S., Satsangi J., Ahmad T., Arnott I.D.R., Bernstein C.N., Brant S.R., Caprilli R., Colombel J.-F., Gasche C., Geboes K. (2005). Toward an Integrated Clinical, Molecular and Serological Classification of Inflammatory Bowel Disease: Report of a Working Party of the 2005 Montreal World Congress of Gastroenterology. Can. J. Gastroenterol..

[12] Satsangi J., Silverberg M.S., Vermeire S., Colombel J. (2006). The Montreal Classification of Inflammatory Bowel Disease: Controversies, Consensus, and Implications. Gut.

[13] Chamouard P., Richert Z., Meyer N., Rahmi G., Baumann R. (2006). Diagnostic Value of C-Reactive Protein for Predicting Activity Level of Crohn’s Disease. Clin. Gastroenterol. Hepatol..

[14] Mosli M.H., Zou G., Garg S.K., Feagan S.G., MacDonald J.K., Chande N., Sandborn W.J., Feagan B.G. (2015). C-Reactive Protein, Fecal Calprotectin, and Stool Lactoferrin for Detection of Endoscopic Activity in Symptomatic Inflammatory Bowel Disease Patients: A Systematic Review and Meta-Analysis. Am. J. Gastroenterol..

[15] Chow D.K.L., Leong R.W.L., Lai L.H., Wong G.L.H., Leung W.-K., Chan F.K.L., Sung J.J.Y. (2008). Changes in Crohn’s Disease Phenotype over Time in the Chinese Population: Validation of the Montreal Classification System. Inflamm. Bowel. Dis..

[16] Torres U.d.S., Rodrigues J.O., Junqueira M.S.G., Uezato S., Netinho J.G. (2010). The Montreal Classification for Crohn’s Disease: Clinical Application to a Brazilian Single-Center Cohort of 90 Consecutive Patients. Arq. Gastroenterol..

[17] Cosnes J., Cattan S., Blain A., Beaugerie L., Carbonnel F., Parc R., Gendre J.-P. (2002). Long-Term Evolution of Disease Behavior of Crohn’s Disease. Inflamm. Bowel. Dis..

[18] Lovasz B.D., Lakatos L., Horvath A., Szita I., Pandur T., Mandel M., Vegh Z., Golovics P.A., Mester G., Balogh M. (2013). Evolution of Disease Phenotype in Adult and Pediatric Onset Crohn’s Disease in a Population-Based Cohort. World J. Gastroenterol..

[19] Louis E., Collard A., Oger A., Degroote E., El Yafi F.A.N., Belaiche J. (2001). Behaviour of Crohn’s Disease According to the Vienna Classification: Changing Pattern over the Course of the Disease. Gut.

[20] Irwin J., Ferguson E., Simms L.A., Hanigan K., Carbonnel F., Radford-Smith G. (2017). A Rolling Phenotype in Crohn’s Disease. PLoS ONE.

[21] Sudhakar P., Verstockt B., Cremer J., Verstockt S., Sabino J., Ferrante M., Vermeire S. (2020). Understanding the Molecular Drivers of Disease Heterogeneity in Crohn’s Disease Using Multi-Omic Data Integration and Network Analysis. Inflamm. Bowel. Dis..

[22] Atreya R., Siegmund B. (2021). Location Is Important: Differentiation between Ileal and Colonic Crohn’s Disease. Nat. Rev. Gastroenterol. Hepatol..

[23] Weiser M., Simon J.M., Kochar B., Tovar A., Israel J.W., Robinson A., Gipson G.R., Schaner M.S., Herfarth H.H., Sartor R.B. (2018). Molecular Classification of Crohn’s Disease Reveals Two Clinically Relevant Subtypes. Gut.

[24] Lin S.-N., Zheng D.-P., Qiu Y., Zhang S.-H., He Y., Chen B.-L., Zeng Z.-R., Mao R., Chen M.-H. (2020). Classifying Crohn’s Disease into Colon-Involving versus Non-Colon-Involving Groups Is a Better Predictor of Clinical Outcomes than the Montreal Classification. Therap. Adv. Gastroenterol..

[25] Durko Ł., Stasikowska-Kanicka O.A., Wagrowska-Danilewicz M., Danilewicz M., Małecka-Panas E.I. (2013). An Analysis of the Correlation of Clinical, Endoscopic and Histological Classifications in Crohn’s Disease. Prz. Gastroenterol..

[26] Weng J., Lin X., Chen X., Liang Y., Xu Y., Cai J., Lu P., Rong Y., Zou Y., Zhu L. (2022). Crohn’s Disease Patients with L4-Esophagogastroduodenal Phenotype Is Associated with a Better Prognosis: A Retrospective Cohort Study. Front. Pharmacol..

[27] Mazor Y., Maza I., Kaufman E., Ben-Horin S., Karban A., Chowers Y., Eliakim R. (2011). Prediction of Disease Complication Occurrence in Crohn’s Disease Using Phenotype and Genotype Parameters at Diagnosis. J. Crohn’s Colitis.

[28] Spekhorst L.M., Visschedijk M.C., Alberts R., Festen E.A., van der Wouden E.-J., Dijkstra G., Weersma R.K. (2014). Performance of the Montreal Classification for Inflammatory Bowel Diseases. World J. Gastroenterol..

[29] Lo B., Vind I., Vester-Andersen M.K., Burisch J. (2020). Validation of Ulcerative Colitis and Crohn’s Disease and Their Phenotypes in the Danish National Patient Registry Using a Population-Based Cohort. Scand J. Gastroenterol..

[30] Gradishar W.J., Moran M.S., Abraham J., Aft R., Agnese D., Allison K.H., Anderson B., Burstein H.J., Chew H., Dang C. (2022). Breast Cancer, Version 3.2022, NCCN Clinical Practice Guidelines in Oncology. J. Natl. Compr. Canc. Netw..

[31] Vermeire S., Van Assche G., Rutgeerts P. (2012). Classification of Inflammatory Bowel Disease: The Old and the New. Curr. Opin. Gastroenterol..

[32] Ferrante M., Henckaerts L., Joossens M., Pierik M., Joossens S., Dotan N., Norman G.L., Altstock R.T., Van Steen K., Rutgeerts P. (2007). New Serological Markers in Inflammatory Bowel Disease Are Associated with Complicated Disease Behaviour. Gut.

[33] Dotan I., Fishman S., Dgani Y., Schwartz M., Karban A., Lerner A., Weishauss O., Spector L., Shtevi A., Altstock R.T. (2006). Antibodies against Laminaribioside and Chitobioside Are Novel Serologic Markers in Crohn’s Disease. Gastroenterology.

[34] Vasiliauskas E., Kam L., Karp L., Gaiennie J., Yang H., Targan S. (2000). Marker Antibody Expression Stratifies Crohn’s Disease into Immunologically Homogeneous Subgroups with Distinct Clinical Characteristics. Gut.

[35] Arnott I.D.R., Landers C.J., Nimmo E.J., Drummond H.E., Smith B.K.R., Targan S.R., Satsangi J. (2004). Sero-Reactivity to Microbial Components in Crohn’s Disease Is Associated with Disease Severity and Progression, but Not NOD2/CARD15 Genotype. Am. J. Gastroenterol..

[36] Prideaux L., De Cruz P., Ng S.C., Kamm M.A. (2012). Serological Antibodies in Inflammatory Bowel Disease: A Systematic Review. Inflamm. Bowel Dis..

[37] Mow W.S., Vasiliauskas E.A., Lin Y.-C., Fleshner P.R., Papadakis K.A., Taylor K.D., Landers C.J., Abreu-Martin M.T., Rotter J.I., Yang H. (2004). Association of Antibody Responses to Microbial Antigens and Complications of Small Bowel Crohn’s Disease. Gastroenterology.

[38] Targan S.R., Landers C.J., Yang H., Lodes M.J., Cong Y., Papadakis K.A., Vasiliauskas E., Elson C.O., Hershberg R.M. (2005). Antibodies to CBir1 Flagellin Define a Unique Response That Is Associated Independently with Complicated Crohn’s Disease. Gastroenterology.

[39] Zholudev A., Zurakowski D., Young W., Leichtner A., Bousvaros A. (2004). Serologic Testing with ANCA, ASCA, and Anti-OmpC in Children and Young Adults with Crohn’s Disease and Ulcerative Colitis: Diagnostic Value and Correlation with Disease Phenotype. Am. J. Gastroenterol..

[40] Cleynen I., Boucher G., Jostins L., Schumm L.P., Zeissig S., Ahmad T., Andersen V., Andrews J.M., Annese V., Brand S. (2016). Inherited Determinants of Crohn’s Disease and Ulcerative Colitis Phenotypes: A Genetic Association Study. Lancet.

[41] Hugot J.P., Chamaillard M., Zouali H., Lesage S., Cézard J.P., Belaiche J., Almer S., Tysk C., O’Morain C.A., Gassull M. (2001). Association of NOD2 Leucine-Rich Repeat Variants with Susceptibility to Crohn’s Disease. Nature.

[42] Ogura Y., Bonen D.K., Inohara N., Nicolae D.L., Chen F.F., Ramos R., Britton H., Moran T., Karaliuskas R., Duerr R.H. (2001). A Frameshift Mutation in NOD2 Associated with Susceptibility to Crohn’s Disease. Nature.

[43] Ahmad T., Armuzzi A., Bunce M., Mulcahy-Hawes K., Marshall S.E., Orchard T.R., Crawshaw J., Large O., de Silva A., Cook J.T. (2002). The Molecular Classification of the Clinical Manifestations of Crohn’s Disease. Gastroenterology.

[44] Jostins L., Ripke S., Weersma R.K., Duerr R.H., McGovern D.P., Hui K.Y., Lee J.C., Schumm L.P., Sharma Y., Anderson C.A. (2012). Host-Microbe Interactions Have Shaped the Genetic Architecture of Inflammatory Bowel Disease. Nature.

[45] de Lange K.M., Moutsianas L., Lee J.C., Lamb C.A., Luo Y., Kennedy N.A., Jostins L., Rice D.L., Gutierrez-Achury J., Ji S.-G. (2017). Genome-Wide Association Study Implicates Immune Activation of Multiple Integrin Genes in Inflammatory Bowel Disease. Nat. Genet..

[46] Sazonovs A., Stevens C.R., Venkataraman G.R., Yuan K., Avila B., Abreu M.T., Ahmad T., Allez M., Ananthakrishnan A.N., Atzmon G. (2022). Large-Scale Sequencing Identifies Multiple Genes and Rare Variants Associated with Crohn’s Disease Susceptibility. Nat. Genet..

[47] Cortes A., Brown M.A. (2011). Promise and Pitfalls of the Immunochip. Arthritis Res Ther.

[48] Wray N.R., Lin T., Austin J., McGrath J.J., Hickie I.B., Murray G.K., Visscher P.M. (2021). From Basic Science to Clinical Application of Polygenic Risk Scores: A Primer. JAMA Psychiatry.

[49] Abakkouy Y., Cleynen I. (2021). The Promise of Polygenic Risk Scores as a Research Tool to Analyse the Genetics Underlying IBD Phenotypes. J. Crohns Colitis.

[50] Voskuil M.D., Spekhorst L.M., van der Sloot K.W.J., Jansen B.H., Dijkstra G., van der Woude C.J., Hoentjen F., Pierik M.J., van der Meulen A.E., de Boer N.K.H. (2021). Genetic Risk Scores Identify Genetic Aetiology of Inflammatory Bowel Disease Phenotypes. J. Crohns Colitis.

[51] Lee J.C., Biasci D., Roberts R., Gearry R.B., Mansfield J.C., Ahmad T., Prescott N.J., Satsangi J., Wilson D.C., Jostins L. (2017). Genome-Wide Association Study Identifies Distinct Genetic Contributions to Prognosis and Susceptibility in Crohn’s Disease. Nat. Genet..

[52] Lawrance I.C., Fiocchi C., Chakravarti S. (2001). Ulcerative Colitis and Crohn’s Disease: Distinctive Gene Expression Profiles and Novel Susceptibility Candidate Genes. Hum. Mol. Genet..

[53] Burczynski M.E., Peterson R.L., Twine N.C., Zuberek K.A., Brodeur B.J., Casciotti L., Maganti V., Reddy P.S., Strahs A., Immermann F. (2006). Molecular Classification of Crohn’s Disease and Ulcerative Colitis Patients Using Transcriptional Profiles in Peripheral Blood Mononuclear Cells. J. Mol. Diagn..

[54] Hong S.N., Joung J.-G., Bae J.S., Lee C.S., Koo J.S., Park S.J., Im J.P., Kim Y.S., Kim J.W., Park W.Y. (2017). RNA-Seq Reveals Transcriptomic Differences in Inflamed and Noninflamed Intestinal Mucosa of Crohn’s Disease Patients Compared with Normal Mucosa of Healthy Controls. Inflamm. Bowel. Dis..

[55] Kaser A., Ludwiczek O., Holzmann S., Moschen A.R., Weiss G., Enrich B., Graziadei I., Dunzendorfer S., Wiedermann C.J., Mürzl E. (2004). Increased Expression of CCL20 in Human Inflammatory Bowel Disease. J. Clin. Immunol..

[56] Dobre M., Milanesi E., Mănuc T.E., Arsene D.E., Ţieranu C.G., Maj C., Becheanu G., Mănuc M. (2018). Differential Intestinal Mucosa Transcriptomic Biomarkers for Crohn’s Disease and Ulcerative Colitis. J. Immunol. Res..

[57] Martin J.C., Chang C., Boschetti G., Ungaro R., Giri M., Grout J.A., Gettler K., Chuang L.-S., Nayar S., Greenstein A.J. (2019). Single-Cell Analysis of Crohn’s Disease Lesions Identifies a Pathogenic Cellular Module Associated with Resistance to Anti-TNF Therapy. Cell.

[58] Kong L., Pokatayev V., Lefkovith A., Carter G.T., Creasey E.A., Krishna C., Subramanian S., Kochar B., Ashenberg O., Lau H. (2023). The Landscape of Immune Dysregulation in Crohn’s Disease Revealed through Single-Cell Transcriptomic Profiling in the Ileum and Colon. Immunity.

[59] Burke J.P., Ferrante M., Dejaegher K., Watson R.W.G., Docherty N.G., De Hertogh G., Vermeire S., Rutgeerts P., D’Hoore A., Penninckx F. (2008). Transcriptomic Analysis of Intestinal Fibrosis-Associated Gene Expression in Response to Medical Therapy in Crohn’s Disease. Inflamm. Bowel. Dis..

[60] Arnauts K., Verstockt B., Ramalho A.S., Vermeire S., Verfaillie C., Ferrante M. (2020). Ex Vivo Mimicking of Inflammation in Organoids Derived From Patients With Ulcerative Colitis. Gastroenterology.

[61] d’Aldebert E., Quaranta M., Sébert M., Bonnet D., Kirzin S., Portier G., Duffas J.-P., Chabot S., Lluel P., Allart S. (2020). Characterization of Human Colon Organoids From Inflammatory Bowel Disease Patients. Front. Cell Dev. Biol..

[62] Niklinska-Schirtz B.J., Venkateswaran S., Anbazhagan M., Kolachala V.L., Prince J., Dodd A., Chinnadurai R., Gibson G., Denson L.A., Cutler D.J. (2021). Ileal Derived Organoids From Crohn’s Disease Patients Show Unique Transcriptomic and Secretomic Signatures. Cell. Mol. Gastroenterol. Hepatol..

[63] Angus H.C.K., Butt A.G., Schultz M., Kemp R.A. (2019). Intestinal Organoids as a Tool for Inflammatory Bowel Disease Research. Front. Med..

[64] Timp W., Timp G. (2020). Beyond Mass Spectrometry, the next Step in Proteomics. Sci. Adv..

[65] Nanni P., Parisi D., Roda G., Casale M., Belluzzi A., Roda E., Mayer L., Roda A. (2007). Serum Protein Profiling in Patients with Inflammatory Bowel Diseases Using Selective Solid-Phase Bulk Extraction, Matrix-Assisted Laser Desorption/Ionization Time-of-Flight Mass Spectrometry and Chemometric Data Analysis. Rapid Commun. Mass Spectrom..

[66] Basso D., Padoan A., D’Incà R., Arrigoni G., Scapellato M.L., Contran N., Franchin C., Lorenzon G., Mescoli C., Moz S. (2020). Peptidomic and Proteomic Analysis of Stool for Diagnosing IBD and Deciphering Disease Pathogenesis. Clin. Chem. Lab. Med. (CCLM).

[67] Klein O., Fogt F., Hollerbach S., Nebrich G., Boskamp T., Wellmann A. (2020). Classification of Inflammatory Bowel Disease from Formalin-Fixed, Paraffin-Embedded Tissue Biopsies via Imaging Mass Spectrometry. Proteomics Clin. Appl..

[68] Starr A.E., Deeke S.A., Ning Z., Chiang C.-K., Zhang X., Mottawea W., Singleton R., Benchimol E.I., Wen M., Mack D.R. (2017). Proteomic Analysis of Ascending Colon Biopsies from a Paediatric Inflammatory Bowel Disease Inception Cohort Identifies Protein Biomarkers That Differentiate Crohn’s Disease from UC. Gut.

[69] Andersson E., Bergemalm D., Kruse R., Neumann G., D’Amato M., Repsilber D., Halfvarson J. (2017). Subphenotypes of Inflammatory Bowel Disease Are Characterized by Specific Serum Protein Profiles. PLoS ONE.

[70] Rukmangadachar L.A., Makharia G.K., Mishra A., Das P., Hariprasad G., Srinivasan A., Gupta S.D., Ahuja V., Acharya S.K. (2016). Proteome Analysis of the Macroscopically Affected Colonic Mucosa of Crohn’s Disease and Intestinal Tuberculosis. Sci. Rep..

[71] Leibovitzh H., Lee S.-H., Raygoza Garay J.A., Espin-Garcia O., Xue M., Neustaeter A., Goethel A., Huynh H.Q., Griffiths A.M., Turner D. (2023). Immune Response and Barrier Dysfunction-Related Proteomic Signatures in Preclinical Phase of Crohn’s Disease Highlight Earliest Events of Pathogenesis. Gut.

[72] Meuwis M.-A., Fillet M., Lutteri L., Marée R., Geurts P., de Seny D., Malaise M., Chapelle J.-P., Wehenkel L., Belaiche J. (2008). Proteomics for Prediction and Characterization of Response to Infliximab in Crohn’s Disease: A Pilot Study. Clin. Biochem..

[73] Hatsugai M., Kurokawa M.S., Kouro T., Nagai K., Arito M., Masuko K., Suematsu N., Okamoto K., Itoh F., Kato T. (2010). Protein Profiles of Peripheral Blood Mononuclear Cells Are Useful for Differential Diagnosis of Ulcerative Colitis and Crohn’s Disease. J. Gastroenterol..

[74] Townsend P., Zhang Q., Shapiro J., Webb-Robertson B.-J., Bramer L., Schepmoes A.A., Weitz K.K., Mallette M., Moniz H., Bright R. (2015). Serum Proteome Profiles in Stricturing Crohn’s Disease: A Pilot Study. Inflamm. Bowel. Dis..

[75] Vitali R., Palone F., Armuzzi A., Fulci V., Negroni A., Carissimi C., Cucchiara S., Stronati L. (2023). Proteomic Analysis Identifies Three Reliable Biomarkers of Intestinal Inflammation in the Stools of Patients With Inflammatory Bowel Disease. J. Crohns Colitis.

[76] D’Haens G., Kelly O., Battat R., Silverberg M.S., Laharie D., Louis E., Savarino E., Bodini G., Yarur A., Boland B.S. (2020). Development and Validation of a Test to Monitor Endoscopic Activity in Patients With Crohn’s Disease Based on Serum Levels of Proteins. Gastroenterology.

[77] Kalla R., Adams A.T., Bergemalm D., Vatn S., Kennedy N.A., Ricanek P., Lindstrom J., Ocklind A., Hjelm F., Ventham N.T. (2021). Serum Proteomic Profiling at Diagnosis Predicts Clinical Course, and Need for Intensification of Treatment in Inflammatory Bowel Disease. J. Crohn’s Colitis.

[78] Lee J.W.J., Plichta D., Hogstrom L., Borren N.Z., Lau H., Gregory S.M., Tan W., Khalili H., Clish C., Vlamakis H. (2021). Multi-Omics Reveal Microbial Determinants Impacting Responses to Biologic Therapies in Inflammatory Bowel Disease. Cell Host. Microbe.

[79] Bourgonje A.R., Hu S., Spekhorst L.M., Zhernakova D.V., Vich Vila A., Li Y., Voskuil M.D., van Berkel L.A., Bley Folly B., Charrout M. (2021). The Effect of Phenotype and Genotype on the Plasma Proteome in Patients with Inflammatory Bowel Disease. J. Crohns Colitis.

[80] van Zalm P.W., Ahmed S., Fatou B., Schreiber R., Barnaby O., Boxer A., Zetterberg H., Steen J.A., Steen H. (2023). Meta-Analysis of Published Cerebrospinal Fluid Proteomics Data Identifies and Validates Metabolic Enzyme Panel as Alzheimer’s Disease Biomarkers. Cell Rep. Med..

[81] Tabb D.L., Vega-Montoto L., Rudnick P.A., Variyath A.M., Ham A.-J.L., Bunk D.M., Kilpatrick L.E., Billheimer D.D., Blackman R.K., Cardasis H.L. (2010). Repeatability and Reproducibility in Proteomic Identifications by Liquid Chromatography—Tandem Mass Spectrometry. J. Proteome Res..

[82] Noble A.J., Nowak J.K., Adams A.T., Uhlig H.H., Satsangi J. (2023). Defining Interactions Between the Genome, Epigenome, and the Environment in Inflammatory Bowel Disease: Progress and Prospects. Gastroenterology.

[83] Ventham N.T., Kennedy N.A., Nimmo E.R., Satsangi J. (2013). Beyond Gene Discovery in Inflammatory Bowel Disease: The Emerging Role of Epigenetics. Gastroenterology.

[84] Wang T., Xia P., Su P. (2022). High-Dimensional DNA Methylation Mediates the Effect of Smoking on Crohn’s Disease. Front. Genet..

[85] Wiklund P., Karhunen V., Richmond R.C., Parmar P., Rodriguez A., De Silva M., Wielscher M., Rezwan F.I., Richardson T.G., Veijola J. (2019). DNA Methylation Links Prenatal Smoking Exposure to Later Life Health Outcomes in Offspring. Clin. Epigenetics.

[86] Vieujean S., Caron B., Haghnejad V., Jouzeau J.-Y., Netter P., Heba A.-C., Ndiaye N.C., Moulin D., Barreto G., Danese S. (2022). Impact of the Exposome on the Epigenome in Inflammatory Bowel Disease Patients and Animal Models. Int. J. Mol. Sci..

[87] Nimmo E.R., Prendergast J.G., Aldhous M.C., Kennedy N.A., Henderson P., Drummond H.E., Ramsahoye B.H., Wilson D.C., Semple C.A., Satsangi J. (2012). Genome-Wide Methylation Profiling in Crohn’s Disease Identifies Altered Epigenetic Regulation of Key Host Defense Mechanisms Including the Th17 Pathway. Inflamm. Bowel Dis..

[88] Hornschuh M., Wirthgen E., Wolfien M., Singh K.P., Wolkenhauer O., Däbritz J. (2021). The Role of Epigenetic Modifications for the Pathogenesis of Crohn’s Disease. Clin. Epigenetics.

[89] Joustra V., Hageman I.L., Satsangi J., Adams A., Ventham N.T., de Jonge W.J., Henneman P., D’Haens G.R., Li Yim A.Y.F. (2023). Systematic Review and Meta-Analysis of Peripheral Blood DNA Methylation Studies in Inflammatory Bowel Disease. J. Crohns Colitis.

[90] Ventham N.T., Kennedy N.A., Adams A.T., Kalla R., Heath S., O’Leary K.R., Drummond H., Wilson D.C., Gut I.G., Nimmo E.R. (2016). Integrative Epigenome-Wide Analysis Demonstrates That DNA Methylation May Mediate Genetic Risk in Inflammatory Bowel Disease. Nat. Commun..

[91] Kalla R., Adams A.T., Nowak J.K., Bergemalm D., Vatn S., Ventham N.T., Kennedy N.A., Ricanek P., Lindstrom J., Söderholm J. (2022). Analysis of Systemic Epigenetic Alterations in Inflammatory Bowel Disease: Defining Geographical, Genetic and Immune-Inflammatory Influences on the Circulating Methylome. J. Crohns Colitis.

[92] Adams A.T., Kennedy N.A., Hansen R., Ventham N.T., O’Leary K.R., Drummond H.E., Noble C.L., El-Omar E., Russell R.K., Wilson D.C. (2014). Two-Stage Genome-Wide Methylation Profiling in Childhood-Onset Crohn’s Disease Implicates Epigenetic Alterations at the VMP1/MIR21 and HLA Loci. Inflamm. Bowel. Dis..

[93] Sadler T., Bhasin J.M., Xu Y., Barnholz-Sloan J., Chen Y., Ting A.H., Stylianou E. (2016). Genome-Wide Analysis of DNA Methylation and Gene Expression Defines Molecular Characteristics of Crohn’s Disease-Associated Fibrosis. Clin. Epigenetics.

[94] Li Yim A.Y.F., de Bruyn J.R., Duijvis N.W., Sharp C., Ferrero E., de Jonge W.J., Wildenberg M.E., Mannens M.M.A.M., Buskens C.J., D’Haens G.R. (2018). A Distinct Epigenetic Profile Distinguishes Stenotic from Non-Inflamed Fibroblasts in the Ileal Mucosa of Crohn’s Disease Patients. PLoS ONE.

[95] Ventham N.T., Kennedy N.A., Kalla R., Adams A.T., Noble A., Ennis H., Mowat C., Dunlop M.G., Satsangi J. (2023). Genome-Wide Methylation Profiling in 229 Patients With Crohn’s Disease Requiring Intestinal Resection: Epigenetic Analysis of the Trial of Prevention of Post-Operative Crohn’s Disease (TOPPIC). Cell. Mol. Gastroenterol. Hepatol..

[96] Howell K.J., Kraiczy J., Nayak K.M., Gasparetto M., Ross A., Lee C., Mak T.N., Koo B.-K., Kumar N., Lawley T. (2018). DNA Methylation and Transcription Patterns in Intestinal Epithelial Cells From Pediatric Patients With Inflammatory Bowel Diseases Differentiate Disease Subtypes and Associate With Outcome. Gastroenterology.

[97] Joustra V., Li Yim A., Hageman I., Levin E., Noble A., Chapman T., McGregor C., Adams A., Satsangi J., de Jonge W. (2023). OP03 Highly Stable Epigenome-Wide Peripheral Blood DNA Methylation Signatures Accurately Predict Endoscopic Response to Adalimumab, Vedolizumab and Ustekinumab in Crohn’s Disease Patients: The EPIC-CD Study. J. Crohn’s Colitis.

[98] Joustra V., Hageman I., Li Yim A., Levin E., Satsangi J., Adams A., De Jonge W., Henneman P., D’Haens G., on behalf of the EPIC consortium (2022). OP29 Peripheral Blood DNA Methylation Biomarkers Accurately Predict Clinical- and Endoscopic Response to Vedolizumab in a Real-Life Cohort of Crohn’s Disease Patients. J. Crohn’s Colitis.

[99] Joustra V., Li Yim A.Y.F., Hageman I., Levin E., Adams A., Satsangi J., de Jonge W.J., Henneman P., D’Haens G. (2022). Long-Term Temporal Stability of Peripheral Blood DNA Methylation Profiles in Patients With Inflammatory Bowel Disease. Cell Mol. Gastroenterol. Hepatol..

[100] Somineni H.K., Venkateswaran S., Kilaru V., Marigorta U.M., Mo A., Okou D.T., Kellermayer R., Mondal K., Cobb D., Walters T.D. (2019). Blood-Derived DNA Methylation Signatures of Crohn’s Disease and Severity of Intestinal Inflammation. Gastroenterology.

[101] Lloyd-Price J., Abu-Ali G., Huttenhower C. (2016). The Healthy Human Microbiome. Genome Med..

[102] Santana P.T., Rosas S.L.B., Ribeiro B.E., Marinho Y., de Souza H.S.P. (2022). Dysbiosis in Inflammatory Bowel Disease: Pathogenic Role and Potential Therapeutic Targets. Int. J. Mol. Sci..

[103] Johnson J.S., Spakowicz D.J., Hong B.-Y., Petersen L.M., Demkowicz P., Chen L., Leopold S.R., Hanson B.M., Agresta H.O., Gerstein M. (2019). Evaluation of 16S RRNA Gene Sequencing for Species and Strain-Level Microbiome Analysis. Nat. Commun..

[104] Pascal V., Pozuelo M., Borruel N., Casellas F., Campos D., Santiago A., Martinez X., Varela E., Sarrabayrouse G., Machiels K. (2017). A Microbial Signature for Crohn’s Disease. Gut.

[105] Abdel-Rahman L.I.H., Morgan X.C. (2023). Searching for a Consensus Among Inflammatory Bowel Disease Studies: A Systematic Meta-Analysis. Inflamm. Bowel. Dis..

[106] Amos G.C.A., Sergaki C., Logan A., Iriarte R., Bannaga A., Chandrapalan S., Wellington E.M.H., Rijpkema S., Arasaradnam R.P. (2021). Exploring How Microbiome Signatures Change across Inflammatory Bowel Disease Conditions and Disease Locations. Sci. Rep..

[107] Gonzalez C.G., Mills R.H., Zhu Q., Sauceda C., Knight R., Dulai P.S., Gonzalez D.J. (2022). Location-Specific Signatures of Crohn’s Disease at a Multi-Omics Scale. Microbiome.

[108] Martinez-Medina M., Aldeguer X., Lopez-Siles M., González-Huix F., López-Oliu C., Dahbi G., Blanco J.E., Blanco J., Garcia-Gil L.J., Darfeuille-Michaud A. (2009). Molecular Diversity of Escherichia Coli in the Human Gut: New Ecological Evidence Supporting the Role of Adherent-Invasive E. Coli (AIEC) in Crohn’s Disease. Inflamm. Bowel. Dis..

[109] Kugathasan S., Denson L.A., Walters T.D., Kim M.-O., Marigorta U.M., Schirmer M., Mondal K., Liu C., Griffiths A., Noe J.D. (2017). Prediction of Complicated Disease Course for Children Newly Diagnosed with Crohn’s Disease: A Multicentre Inception Cohort Study. Lancet.

[110] Lopez J., Grinspan A. (2016). Fecal Microbiota Transplantation for Inflammatory Bowel Disease. Gastroenterol. Hepatol..

[111] Knox N.C., Forbes J.D., Van Domselaar G., Bernstein C.N. (2019). The Gut Microbiome as a Target for IBD Treatment: Are We There Yet?. Curr. Treat. Options Gastroenterol..

[112] Patti G.J., Yanes O., Siuzdak G. (2012). Metabolomics: The Apogee of the Omic Triology. Nat. Rev. Mol. Cell Biol..

[113] Griffiths W.J., Koal T., Wang Y., Kohl M., Enot D.P., Deigner H.-P. (2010). Targeted Metabolomics for Biomarker Discovery. Angew. Chem. Int. Ed..

[114] Chetwynd A.J., Dunn W.B., Rodriguez-Blanco G., Sussulini A. (2017). Collection and Preparation of Clinical Samples for Metabolomics. Metabolomics: From Fundamentals to Clinical Applications.

[115] Aldars-García L., Gisbert J.P., Chaparro M. (2021). Metabolomics Insights into Inflammatory Bowel Disease: A Comprehensive Review. Pharmaceuticals.

[116] Marchesi J.R., Holmes E., Khan F., Kochhar S., Scanlan P., Shanahan F., Wilson I.D., Wang Y. (2007). Rapid and Noninvasive Metabonomic Characterization of Inflammatory Bowel Disease. J. Proteome Res..

[117] Gallagher K., Catesson A., Griffin J.L., Holmes E., Williams H.R.T. (2021). Metabolomic Analysis in Inflammatory Bowel Disease: A Systematic Review. J. Crohns Colitis.

[118] Vila A.V., Hu S., Andreu-Sánchez S., Collij V., Jansen B.H., Augustijn H.E., Bolte L.A., Ruigrok R.A.A.A., Abu-Ali G., Giallourakis C. (2023). Faecal Metabolome and Its Determinants in Inflammatory Bowel Disease. Gut.

[119] Mossotto E., Boberska J., Ashton J.J., Stafford I.S., Cheng G., Baker J., Borca F., Phan H.T.T., Coelho T.F., Beattie R.M. (2022). Evidence of a Genetically Driven Metabolomic Signature in Actively Inflamed Crohn’s Disease. Sci. Rep..

[120] Xu X., Ocansey D.K.W., Hang S., Wang B., Amoah S., Yi C., Zhang X., Liu L., Mao F. (2022). The Gut Metagenomics and Metabolomics Signature in Patients with Inflammatory Bowel Disease. Gut Pathogens.

[121] Shimizu T. (2009). Lipid Mediators in Health and Disease: Enzymes and Receptors as Therapeutic Targets for the Regulation of Immunity and Inflammation. Annu. Rev. Pharmacol. Toxicol..

[122] Shores D.R., Binion D.G., Freeman B.A., Baker P.R.S. (2011). New Insights into the Role of Fatty Acids in the Pathogenesis and Resolution of Inflammatory Bowel Disease. Inflamm. Bowel. Dis..

[123] Fahy E., Cotter D., Sud M., Subramaniam S. (2011). Lipid Classification, Structures and Tools. Biochim. Biophys. Acta.

[124] Vale G., Martin S.A., Mitsche M.A., Thompson B.M., Eckert K.M., McDonald J.G. (2019). Three-Phase Liquid Extraction: A Simple and Fast Method for Lipidomic Workflows. J. Lipid Res..

[125] Cajka T., Fiehn O. (2014). Comprehensive Analysis of Lipids in Biological Systems by Liquid Chromatography-Mass Spectrometry. Trends Analyt. Chem..

[126] Manfredi M., Conte E., Barberis E., Buzzi A., Robotti E., Caneparo V., Cecconi D., Brandi J., Vanni E., Finocchiaro M. (2019). Integrated Serum Proteins and Fatty Acids Analysis for Putative Biomarker Discovery in Inflammatory Bowel Disease. J. Proteom..

[127] Fan F., Mundra P.A., Fang L., Galvin A., Moore X.L., Weir J.M., Wong G., White D.A., Chin-Dusting J., Sparrow M.P. (2015). Lipidomic Profiling in Inflammatory Bowel Disease: Comparison Between Ulcerative Colitis and Crohn’s Disease. Inflamm. Bowel. Dis..

[128] Iwatani S., Iijima H., Otake Y., Amano T., Tani M., Yoshihara T., Tashiro T., Tsujii Y., Inoue T., Hayashi Y. (2020). Novel Mass Spectrometry-Based Comprehensive Lipidomic Analysis of Plasma from Patients with Inflammatory Bowel Disease. J. Gastroenterol. Hepatol..

[129] Brown E.M., Ke X., Hitchcock D., Jeanfavre S., Avila-Pacheco J., Nakata T., Arthur T.D., Fornelos N., Heim C., Franzosa E.A. (2019). Bacteroides-Derived Sphingolipids Are Critical for Maintaining Intestinal Homeostasis and Symbiosis. Cell Host. Microbe.

[130] Guan S., Jia B., Chao K., Zhu X., Tang J., Li M., Wu L., Xing L., Liu K., Zhang L. (2020). UPLC-QTOF-MS-Based Plasma Lipidomic Profiling Reveals Biomarkers for Inflammatory Bowel Disease Diagnosis. J. Proteome Res..

[131] Scoville E.A., Allaman M.M., Brown C.T., Motley A.K., Horst S.N., Williams C.S., Koyama T., Zhao Z., Adams D.W., Beaulieu D.B. (2018). Alterations in Lipid, Amino Acid, and Energy Metabolism Distinguish Crohn’s Disease from Ulcerative Colitis and Control Subjects by Serum Metabolomic Profiling. Metabolomics.

[132] Jansson J., Willing B., Lucio M., Fekete A., Dicksved J., Halfvarson J., Tysk C., Schmitt-Kopplin P. (2009). Metabolomics Reveals Metabolic Biomarkers of Crohn’s Disease. PLoS ONE.

[133] Tefas C., Ciobanu L., Tanțău M., Moraru C., Socaciu C. (2020). The Potential of Metabolic and Lipid Profiling in Inflammatory Bowel Diseases: A Pilot Study. Bosn. J. Basic Med. Sci..

[134] Horta D., Moreno-Torres M., Ramírez-Lázaro M.J., Lario S., Kuligowski J., Sanjuan-Herráez J.D., Quintas G., Villoria A., Calvet X. (2021). Analysis of the Association between Fatigue and the Plasma Lipidomic Profile of Inflammatory Bowel Disease Patients. J. Proteome Res..

[135] Lee Y., Choo J., Kim S.J., Heo G., Pothoulakis C., Kim Y.-H., Im E. (2017). Analysis of Endogenous Lipids during Intestinal Wound Healing. PLoS ONE.

[136] Wang R., Gu X., Dai W., Ye J., Lu F., Chai Y., Fan G., Gonzalez F.J., Duan G., Qi Y. (2016). A Lipidomics Investigation into the Intervention of Celastrol in Experimental Colitis. Mol. Biosyst..

[137] Cecerska-Heryć E., Ronkowski B., Heryć R., Serwin N., Grygorcewicz B., Roszak M., Galant K., Dołęgowska B. (2023). Proteomic and Lipidomic Biomarkers in the Diagnosis and Progression of Inflammatory Bowel Disease—A Review. Proteom. Clin. Appl..

[138] Lee E.G., Yoon Y.C., Yoon J., Lee S.J., Oh Y.-K., Kwon S.W. (2021). Systematic Review of Recent Lipidomics Approaches Toward Inflammatory Bowel Disease. Biomol. Ther..

[139] Ahluwalia B., Moraes L., Magnusson M.K., Öhman L. (2018). Immunopathogenesis of Inflammatory Bowel Disease and Mechanisms of Biological Therapies. Scand. J. Gastroenterol..

[140] Verdier J., Begue B., Cerf-Bensussan N., Ruemmele F.M. (2012). Compartmentalized Expression of Th1 and Th17 Cytokines in Pediatric Inflammatory Bowel Diseases. Inflamm. Bowel. Dis..

[141] Lee H.B., Kim J.H., Yim C.Y., Kim D.G., Ahn D.S. (1997). Differences in Immunophenotyping of Mucosal Lymphocytes between Ulcerative Colitis and Crohn’s Disease. Korean J. Intern. Med..

[142] Kosoy R., Kim-Schulze S., Rahman A., Friedman J.R., Huang R., Peters L.A., Amir E.-A., Perrigoue J., Stojmirovic A., Song W.-M. (2021). Deep Analysis of the Peripheral Immune System in IBD Reveals New Insight in Disease Subtyping and Response to Monotherapy or Combination Therapy. Cell Mol. Gastroenterol. Hepatol..

[143] Kredel L.I., Jödicke L.J., Scheffold A., Gröne J., Glauben R., Erben U., Kühl A.A., Siegmund B. (2019). T-Cell Composition in Ileal and Colonic Creeping Fat—Separating Ileal from Colonic Crohn’s Disease. J. Crohns Colitis.

[144] Levitte S., Jhun I., Neighbors M., Peale F., Grimbaldeston M., Haileselassie Y., Habtezion A., Park K., Rogalla S. (2022). Quantitative immunohistochemical analysis of immune cells reveals immunophenotypes associated with intestinal fibrosis and postoperative stricture recurrence in crohn’s disease. Gastroenterology.

[145] Smids C., Horjus Talabur Horje C.S., Drylewicz J., Roosenboom B., Groenen M.J.M., van Koolwijk E., van Lochem E.G., Wahab P.J. (2018). Intestinal T Cell Profiling in Inflammatory Bowel Disease: Linking T Cell Subsets to Disease Activity and Disease Course. J. Crohns Colitis.

[146] McKinney E.F., Lyons P.A., Carr E.J., Hollis J.L., Jayne D.R.W., Willcocks L.C., Koukoulaki M., Brazma A., Jovanovic V., Kemeny D.M. (2010). A CD8+ T Cell Transcription Signature Predicts Prognosis in Autoimmune Disease. Nat. Med..

[147] Lee J.C., Lyons P.A., McKinney E.F., Sowerby J.M., Carr E.J., Bredin F., Rickman H.M., Ratlamwala H., Hatton A., Rayner T.F. (2011). Gene Expression Profiling of CD8+ T Cells Predicts Prognosis in Patients with Crohn Disease and Ulcerative Colitis. J. Clin. Invest..

[148] Noor N., Brezina B., Negro J.D.L.R., Dowling F., Bond S., Whitehead L., Lee J., Lyons P., McKinney E., Smith K. (2022). Predicting Outcomes for Crohn’s Disease Using a Molecular Biomarker: Profile Trial. Clin. Med..

[149] Parkes M., Noor N.M., Dowling F., Leung H., Bond S., Whitehead L., Upponi S., Kinnon P., Sandham A.P., Lyons P.A. (2018). PRedicting Outcomes For Crohn’s DIsease Using a MoLecular BiomarkEr (PROFILE): Protocol for a Multicentre, Randomised, Biomarker-Stratified Trial. BMJ Open.

[150] Gómez-Cebrián N., Domingo-Ortí I., Poveda J.L., Vicent M.J., Puchades-Carrasco L., Pineda-Lucena A. (2021). Multi-Omic Approaches to Breast Cancer Metabolic Phenotyping: Applications in Diagnosis, Prognosis, and the Development of Novel Treatments. Cancers.

[151] Babu M., Snyder M. (2023). Multi-Omics Profiling for Health. Mol. Cell Proteom..

